# Development and validation of questionnaire-based machine learning models for predicting all-cause mortality in a representative population of China

**DOI:** 10.3389/fpubh.2023.1033070

**Published:** 2023-01-27

**Authors:** Ziyi Li, Na Yang, Liyun He, Jialu Wang, Fan Ping, Wei Li, Lingling Xu, Huabing Zhang, Yuxiu Li

**Affiliations:** Key Laboratory of Endocrinology of National Health Commission, Department of Endocrinology, Translation Medicine Center, Peking Union Medical College Hospital, Chinese Academy of Medical Sciences and Peking Union Medical College, Beijing, China

**Keywords:** mortality, machine learning, prediction model, personalized prediction, questionnaire-based

## Abstract

**Background:**

Considering that the previously developed mortality prediction models have limited applications to the Chinese population, a questionnaire-based prediction model is of great importance for its accuracy and convenience in clinical practice.

**Methods:**

Two national cohort, namely, the China Health and Nutrition Survey (8,355 individual older than 18) and the China Health and Retirement Longitudinal Study (12,711 individuals older than 45) were used for model development and validation. One hundred and fifty-nine variables were compiled to generate predictions. The Cox regression model and six machine learning (ML) models were used to predict all-cause mortality. Finally, a simple questionnaire-based ML prediction model was developed using the best algorithm and validated.

**Results:**

In the internal validation set, all the ML models performed better than the traditional Cox model in predicting 6-year mortality and the random survival forest (RSF) model performed best. The questionnaire-based ML model, which only included 20 variables, achieved a C-index of 0.86 (95%CI: 0.80–0.92). On external validation, the simple questionnaire-based model achieved a C-index of 0.82 (95%CI: 0.77–0.87), 0.77 (95%CI: 0.75–0.79), and 0.79 (95%CI: 0.77–0.81), respectively, in predicting 2-, 9-, and 11-year mortality.

**Conclusions:**

In this prospective population-based study, a model based on the RSF analysis performed best among all models. Furthermore, there was no significant difference between the prediction performance of the questionnaire-based ML model, which only included 20 variables, and that of the model with all variables (including laboratory variables). The simple questionnaire-based ML prediction model, which needs to be further explored, is of great importance for its accuracy and suitability to the Chinese general population.

## Introduction

Accurate identification of individuals at high risk of mortality is valuable for both medical care and public health policymaking (1). Recently, a few prognostic indices and prediction models with variable predictors have been developed to predict an individual's probability of death (2–6). However, most of the current mortality prediction models have been developed using data from the United States and Western Europe (5–7), which may greatly limit their validity for Chinese. With the change of lifestyle in recent years, the number of premature deaths caused by non-communicable diseases has increased. However, the limited mortality prediction research on Chinese mostly focuses on elderly or those suffering from specific diseases, which may not be suitable for the general population (8–10). Prediction of young people's death is also noteworthy for early death of youngers brings more losses to individuals, families, and society. The need for laboratory test data is another reason that limits the application of the current mortality prediction models (2). As a country with a large population and lack of medical resources, the death prediction model based on questionnaire may be more valuable. Thus, developing a questionnaire-based all-cause mortality prediction model for the general population of China is of great significance.

Traditionally, mortality prediction models have only applied logistic or Cox regression models on limited variables, resulting in low predictive performance (6, 11). Over the past decades, many advanced machine learning (ML) techniques have further expanded the traditional medical prediction owing to their ability in processing large-scale data and identifying hidden risk factors of diseases (12). A large number of variables can be simultaneously included in prediction models using ML algorithms. The ability to automatically model non-linear correlations and interactions between different risk factors may allow ML models to outperform statistical models in terms of calibration and discrimination (13, 14). Despite claims that the ML algorithm can revolutionize risk prediction and replace traditional statistical models, the performances of ML algorithms may differ in different areas (15, 16). At present, all-cause mortality prediction models developed using the ML algorithm are still rare. Moreover, studies comparing the performances of different ML algorithms with those of traditional Cox regression models in predicting all-cause mortality are still lacking.

To address these important knowledge gaps, the present study aimed to develop a questionnaire-based mortality prediction model for Chinese by comparing the performances of six ML approaches with that of the commonly used Cox regression model using data from the China Health and Nutrition Survey (CHNS).

## Materials and methods

### Study design and participants

Two national surveys, namely, the CHNS and the China Health and Retirement Longitudinal Study (CHARLS) were used for model development and validation. The details of the CHNS and CHARLS design have been described previously and are discussed in the Supplementary Data (17, 18). Briefly, the CHNS is a national, longitudinal, open cohort study that started in 1989 and has been followed up every 2–4 years. In the 2009 record of the CHNS, the data of subjects included both questionnaire and laboratory variables. The CHARLS is a nationally representative longitudinal survey of the social, economic, and health circumstances of individuals older than 45 in China.

To compare the predictive effect of the laboratory and questionnaire variables on mortality, the model development data of this study were extracted from the 2009 to 2015 survey of the CHNS, which included 12,178 participants. The exclusion criteria were as follows: age < 18 years; no follow-up data; pregnant or breastfeeding; with a history of myocardial infarction, stroke, or any type of tumor; and with more than 30% missing variables. The CHNS cohorts were randomly split into training (80%) and internal validation (20%) datasets to develop the 6-year mortality prediction models. The 2004–2015 CHNS dataset, 2006–2015 CHNS dataset, and the dataset derived from CHARLS were used to externally validate the performance of the model in predicting 11-, 9- and 2-year mortality, separately.

### Mortality ascertainment

The mortality status and date of mortality were confirmed according to the information reported by household members. Years of follow-up were calculated from the time of the baseline to death or censoring in the end survey wave, whichever came first.

### Predictors

All candidate predictors were extracted from the CHNS data. Trained staff administered a standardized questionnaire to collect information on individuals, households, and communities. Trained clinicians performed physical examinations, including measurements of height, weight, and blood pressure. Blood samples were collected from an empty stomach after the participants maintained a regular pattern of life for at least 3 days. The details of all candidate predictors can be found on the following website: http://www.cpc.unc.edu/projects/china/data/datasets. Variables that were missing in more than 30% of the participants were excluded. Ultimately, 159 independent predictors, selected from 11 commonly investigated domains, were measured in the present study. The 11 domains were as follows: (i) demographics, (ii) family relations, (iii) community score, (iv) activity and time spent, (v) socioeconomic status, (vi) macronutrient intakes and dietary behaviors, (vii) lifestyle, (viii) diet and exercise knowledge, (ix) health condition, (x) physical examination, and (xi) laboratory examination. Complete details are provided in the Appendix.

### Development and comparison of models

The Cox regression model and six ML approaches, namely, the least absolute shrinkage and selection operator (LASSO) regression (17), survival tree (19), random survival forest (RSF) (20), conditional inference forest (CIF) (21), boosted generalized linear model (glmBoost) (22), and gradient boosting (GBM) (23, 24), were developed separately to predict the risk of 6-year all-cause mortality. The description of ML algorithms has been described previously and are provided in the Supplementary Data (25, 26). Cross-validation was used to limit mean cross-validation error. To evaluate the performance of the model with different variables, three different sets of variables were separately compiled to generate predictions. The three sets of variables included the 159 variables, all laboratory variables, and all variables except laboratory variables. To develop the questionnaire-based prediction model, the performance of the best ML algorithm was further compared with those of models derived using a similar approach: (1) with the top 20 questionnaire variables, (2) with the top 10 questionnaire variables, and (3) with all variables. Each variable was ranked using the variable importance (VIMP) metric in the RSF algorithm (20). To achieve a simple model, the variables that could be directly obtained through questionnaires without calculation were compiled to generate predictions.

### Sensitivity analysis

To assess the generalizability of the ML-based algorithms in predicting long-term mortality, sensitivity analyses were performed on data that were followed up for 9 and 11 years. Two cohorts were recruited separately from the 2004 and 2006 surveys of the CHNS, followed to the 2015 survey. The inclusion and exclusion criteria used in the sensitivity analysis cohorts were the same as those used in the model development cohort. The variables in the two cohorts were all the questionnaire variables, which were the same as those in the model development cohort. All variables were used to develop the Cox regression model and six ML models to separately predict 9- or 11-year all-cause mortality. The performances of all models were compared.

### Model validation

All models were validated in the 2009 CHNS internal validation cohort to predict the risk of 6-year all-cause mortality. The simple questionnaire-based model was further externally validated in three independent cohorts, namely, the 2004–2015 CHNS dataset, 2006–2015 CHNS dataset, and the dataset derived from the CHARLS.

### Statistical analysis

The missing values for each predictor were imputed using an iterative imputation method based on a random forest algorithm (23). The mean ± standard deviation or median with interquartile range was calculated for continuous variables. Totals and percentages were calculated for categorical variables.

The model discriminatory performance was measured using the time-dependent receiver operating characteristic (ROC) curve and Harrell concordance index (C-index) (27, 28). The ROC curve is a statistical tool used to evaluate the discriminative capacity of a diagnostic test. C statistics, ranging from 0.5 to 1, measure the ability of a model to rank patients from high to low risk. The differences between the C-indices of the different models were determined using the DeLong test (29). Calibration of the mortality prediction model was assessed with the Brier score, with a value of Brier score < 0.25 indicating adequate calibration (30, 31).

All analyses were conducted using the R software (version 4.1). The R packages used included “survival,” “gbm,” “grid,” “party,” “pec,” “mboost,” “glmnet,” and “randomForestSRC.” The statistical significance was set at *P* < 0.05.

## Results

### Participants and predictors

A total of 8,355 individuals recruited in the development cohort of 2009–2015 CHNS and 177 participants died during the 6-year follow-up (Supplementary Figure 1, Table 1). The validation cohort of 2006–2015 CHNS, consisted of 8,126 individuals, and 495 died after 9 years of follow-up. The validation cohort of 2004–2015 CHNS, consisted of 8,827 individuals, and 665 died after 11 years of follow-up. The validation cohort of CHARLS consisted of 12,711 individuals, and 262 died during the 2-year follow-up.

**Table 1:** Baseline characteristics of the study sample stratified by mortality status.

	**Total**	**Died**	**Alive**
No. of subjects	8,355	177	8,178
Follow-up, day	1,864 (627)	1,445 (459)	1,873 (627)
Age, year	50.18 (14.65)	67.91 (11.88)	49.79 (14.46)
Men	4,004 (47.92%)	92 (51.98%)	3,912 (47.84%)
Diabetes	213 (2.55%)	11 (6.21%)	202 (2.47%)
Hypertension	997 (11.93%)	50 (28.29%)	947 (11.57%)
Smoking	2,629 (31.47%)	75 (42.37%)	2,554 (31.20%)
Drinking	2,808 (33.61%)	55 (31.07%)	2,753 (33.62%)

Mean ± standard deviation (SD) or median with interquartile range (IQR) were calculated for continuous variables. Totals and percents were calculated for categorical variables. Smoking, individuals still smoke cigarettes now. Drinking, drinking beer or any alcoholic beverage more than once a month.

In total, 159 variables collected at the baseline were considered candidates for developing the models. The descriptive statistics for variables are presented in Supplementary Tables 1, 2.

### Model performance

**Figure 1:**
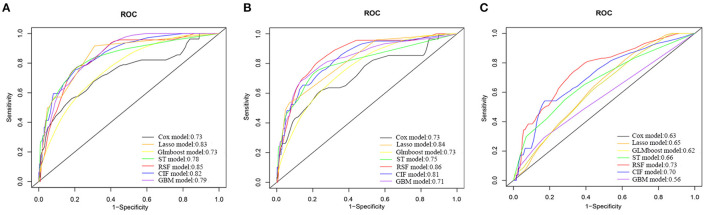
Receiver operating characteristic curves of different models for predicting 6-year all-cause mortality in Internal Validation Cohort. **(A)** Models with all variables. **(B)** Models with variables excluding laboratory variables. **(C)** Models with only laboratory variables. COX, Cox proportional hazards regression model; Lasso, least absolute shrinkage and selection operator regression model; GLMBoost, boosted generalized linear model; ST, survival tree model; CIF, conditional inference forest model; RSF, random forest survival analysis model; GBM, gradient boosting model.

Supplementary Table 3 displays the discrimination and calibration of models in the training cohort. The performance metrics in the internal validation cohort of all models are listed in Table 2. All ML models performed better than the traditional Cox model. The RSF model had the highest C-index [0.86 (95%CI: 0.80–0.92)] and area under the ROC curve (0.85) (Figure 1). And there were significant differences between the C-index of RSF and that of other models which indicating that the RSF model has the best discrimination ability in the present study. The C-indices of models without laboratory variables did not significantly decrease compared with the models with all variables (*p* > 0.05). Compared with the performance of the models that included all variables, those of the models that only included laboratory variables decreased significantly. The ML models demonstrated improved calibration compared with the Cox model (Table 2 and Supplementary Figures 2–4).

**Table 2:** Performance of the models for predicting all-cause mortality in internal validation cohort.

		**COX**	**Lasso**	**glmBoost**	**ST**	**RSF**	**CIF**	**GBM**
All	C-index	0.72 (0.64–0.80)	0.82 (0.75–0.89)	0.82 (0.74–0.88)	0.79 (0.70–0.88)	0.86 (0.80–0.92)	0.82 (0.71–0.93)	0.83 (0.76–0.90)
	Time-ROC	0.73	0.83	0.73	0.78	0.85	0.82	0.79
	Brier	0.12	0.08	0.08	0.08	0.07	0.06	0.07
Without laboratory variables	C-index	0.73 (0.64–0.82)	0.82 (0.74–0.90)	0.82 (0.76–0.88)	0.78 (0.74–0.82)	0.86 (0.79–0.92)	0.82 (0.75–0.89)	0.80 (0.74–0.86)
	Time-ROC	0.73	0.84	0.73	0.75	0.86	0.81	0.81
	Brier	0.12	0.08	0.07	0.08	0.06	0.08	0.08
Laboratory variables	C-index	0.63 (0.53–0.73)	0.66 (0.56–0.76)	0.63 (0.54–0.72)	0.66 (0.57–0.75)	0.72 (0.65–0.79)	0.70 (0.62–0.78)	0.69 (0.61–0.77)
	Time-ROC	0.63	0.65	0.62	0.66	0.73	0.70	0.56
	Brier	0.15	0.15	0.13	0.15	0.12	0.12	0.12

COX, Cox proportional hazards regression model; Lasso, least absolute shrinkage and selection operator regression model; glmBoost, boosted generalized linear model; ST, survival tree model; CIF, conditional inference forest model; RSF, random forest survival analysis model; GBM, gradient boosting model. All: Models with all variables; Without laboratory variables: Models with variables excluding laboratory variables; Laboratory variables: Models with only laboratory variables.

### Sensitivity analyses

In the dataset of the 2006–2015 CHNS, the best performer was the RSF model, which had the highest C-index value 0.85 (95%CI: 0.82–0.88), and the C-index for the Cox regression model was only 0.79 for estimation of the risk of 9-year all-cause mortality. Similarly, the RSF algorithm had the highest C-index value, and the Cox model had the lowest C-index when predicting the 11-year all-cause mortality in the dataset of the 2004–2015 CHNS (Supplementary Tables 4, 5).

### Derivation of questionnaire-based ML models

Considering its best performance among all the algorithms, the RSF algorithm was used to develop questionnaire-based models. Supplementary Figure 5 and Supplementary Table 6 show the relative importance of the top 20 variables in the RSF model (without laboratory variables). The C-index for the RSF with the top 20 self-reported predictor model was 0.86 (95%CI: 0.80–0.92), which was higher than the model with 10 variables. Meanwhile, the discrimination and calibration of the RSF with the top 20 self-reported predictor model was similar to that of the RSF model with all predictors (Table 3 and Supplementary Figure 5). To improve the clinical utility of the prediction model, we implemented the simple ML model which included 20 variables on a publicly available website (https://chnsmortalityprediction.shinyapps.io/mortality-20/) (Supplementary Figure 7). Furthermore, simple ML model which included 10 variables was also implemented on a publicly available website (https://chnsmortalityprediction.shinyapps.io/mortality-10/) (Supplementary Figure 8).

**Table 3:** Performance of the RSF models in the internal validation cohort.

	**All predictors**	**Self-reported top 10 predictors**	**Self-reported top 20 predictors**	**Laboratory variables**
C-index	0.86 (0.80–0.92)	0.84 (0.76–0.92)	0.86 (0.80–0.92)	0.72 (0.65–0.79)
Time-ROC	0.85	0.83	0.84	0.73
Brier	0.07	0.06	0.06	0.12

Data presented are C-index values (95% CIs). The following models were analyzed: RSF with all 159 laboratory variables, RSF with self-reported predictors among top 20/10 variables.

### External validation of the questionnaire-based ML prediction model

In the present study, three external cohorts were used to validate the questionnaire-based model. In the CHNS 2004–2015 cohort, the overall C-index for 11-year mortality was 0.79 (95%CI: 0.77–0.81) and the time-dependent ROC value was 0.81. The overall C-index for 9-year mortality in the CHNS 2006–2015 cohort was 0.77 (95%CI: 0.75–0.79) and the time-dependent ROC value was 0.78. The overall C-index for 2-year mortality in the CHARLS was 0.82 (95%CI: 0.77–0.87). The calibration of all three external validations was acceptable, with a Brier score of < 0.6 (Table 4 and Supplementary Figures 9–11).

**Table 4:** Performance of the predictive model on the external validation cohorts.

	**C-index**	**Time-ROC**	**Brier**
CHARLS	0.82 (0.77–0.87)	0.83	0.06
CHNS (2004–2015)	0.79 (0.77–0.81)	0.81	0.04
CHNS (2006–2015)	0.77 (0.75–0.79)	0.78	0.05

CHARLS, China Health and Retirement Longitudinal Study. CHNS, China Health and Nutrition Survey.

## Discussion

In the present study, we compared the performances of six ML approaches with that of the commonly used Cox proportional hazards regression model for predicting all-cause mortality in Chinese and developed a questionnaire-based ML prediction model based on the best-performing approach. Several key inferences can be drawn from the results. First, ML algorithms can improve traditional approaches used for creating all-cause mortality prediction tools, and the RSF model has the best performance among all models in the present study. Furthermore, the performance of the model based on only 20 questionnaire variables is similar to that of the model with all variables which included laboratory variables. Finally, the questionnaire-based ML model developed from the CHNS data can estimate all-cause mortality with good discrimination for Chinese.

To limit the burden on already strained health systems and improve efficiency, identifying individuals with a high risk of death to provide more targeted prevention and treatment is of great significance. Most of the available mortality prediction models are constructed using traditional statistical techniques (2, 3, 5, 7, 10, 32). Because of the limited variables included and the simple model fitting method, the performances of the prediction models constructed using traditional methods may be greatly limited. Recently, ML has gradually attracted interest because of its powerful ability to identify potential variables and fit models (33–36). Many studies have found that prediction models constructed using ML algorithms have higher performances than traditional statistical methods in predicting the occurrence of clinical events, such as hypoglycemia and cardiovascular events, and prognosis of tumors (20, 37–39). However, it is worth noting that ML techniques are not always better than “classical” statistical methods (40). The effectiveness of ML algorithms on specific data and problems must be evaluated separately. Recently, some studies on the application of ML in determining all-cause mortality have been conducted (41–45). However, most of these studies regarded death as a binary event rather than a time-to-event survival outcome. Directly applying popular ML models to data without censoring substantially biases risk predictions (16). To the best of our knowledge, there is no comprehensive comparative study between ML and traditional methods on the premise of taking death as a time-to-event outcome. In the present study, six ML algorithms derived from three major approaches to predict survival events were compared with the traditional Cox model in the prediction of all-cause mortality. The results indicated that all ML methods performed better than the traditional Cox model. This finding suggests that ML algorithms can improve traditional approaches if the same data are used for mortality prediction. Meanwhile, the RSF model has the highest C-index, which is higher than those of the CIF and GBD models, indicating that a complex model does not necessarily result in a higher predictive power.

Another important advantage of ML algorithms is that they rely on machine-guided data-driven methods instead of experience-guided data analysis to identify risk factors and generate the best fit for the data. Traditional mortality prediction models only empirically incorporate a limited number of variables. Even if more factors are included, the non-linear relationship cannot be identified because of the characteristics of the algorithm. Therefore, it is possible to ignore factors that have important impacts on death. ML algorithms have great advantages in dealing with a wide range of complex datasets with multifactor causality and potentially non-intuitive interactions (46). In the present study, we used an ML algorithm to incorporate 159 factors and explored the potential factors that might affect death. The results lay a foundation for further exploration of the risk factors of all-cause mortality.

Recently, many clinical prediction models have been developed. However, the applications of these tools are limited. One major reason is the inclusion of laboratory variables in the current prediction models (4, 5). Simple questionnaire questions may be closely related to all-cause mortality. Recently, there have been a few questionnaire-based all-cause mortality prediction model which the C-indexes of model is higher than 0.7 (4, 5). However, these prediction models are established by traditional COX model. At present, there is no questionnaire-based all-cause mortality prediction model based on machine learning algorithm for the general population in China. In the present study, there is no significant difference between ML-based models with and without laboratory variables. There may be several reasons for this. First, although laboratory variables, such as glucose, are closely related with disease which are associated with mortality, the level of laboratory variable included in the study cannot fully reflect the situation of diseases for that patients might have normal level of laboratory variable during treatment. Second, the laboratory examination indicators involved in our study only include basic blood indexes (such as glucose, red blood cell count, hemoglobin, index for lipid metabolism). The prediction efficiency of a single variable may be limited in the general population when predicting all-cause mortality. Therefore, the prediction effect of basic blood indicators involved in our study on all-cause mortality may be limited. Previous studies indicated that the c-index for predicting all-cause mortality using basic blood indicators only reached 0.7 (2, 47). Third, in terms of predicting all-cause death, the prediction ability of socioeconomic indicators, lifestyle and disease history is not weaker than that of basic blood indicators (7). Consistent with previous reports, the present results in our study suggest that the prediction performance of a model may not be significantly improved by further adding laboratory variables when sufficient variables are included in the process of fitting the model with an ML algorithm. To reduce the complexity of the model and increase its clinical practicability, we further developed a simple prediction model that only included 20 variables. The performance of the simple questionnaire-based ML model was not significantly different from those of models with all variables. To further improve the application of the model, a web page for our simple questionnaire-based ML model was developed. Therefore, anyone can use this ML-based model to improve their self-awareness of health status.

Another important factor that affects model application is the development data of the model. No predictive model can be used in clinical practice if its effectiveness is not yet tested in other populations (48). The prediction model developed for a certain population may not achieve a good performance when applied to other populations. For example, the performance of a model developed using data from the United States may deteriorate when it is applied to Britons (49). Therefore, most of the available mortality prediction models developed using data from European and American countries may not be suitable for China because of the large differences in lifestyle, culture, and genes (4, 25, 32). In this mortality prediction model study, to develop a prediction model for Chinese, data from the CHNS, which comprehensively contain information on diet, exercise, and lifestyle of Chinese, were used. To the best of our knowledge, previous all-cause mortality prediction models for Chinese have mostly focused on specific groups, such as the elderly and those with diabetes (8–10). There is no all-cause mortality prediction model for the general population in China. In this study, we used data from all adults to predict all-cause mortality for the general population. More importantly, our prediction model was further validated using external datasets. Owing to the limited follow-up time and size of the cohort, the number of deaths in the development cohort of the questionnaire-based model was small, which might have influenced the accuracy of the model. To further evaluate the effectiveness of the questionnaire-based model, cohorts with longer follow-up times and larger number of deaths were used as external validation datasets. The questionnaire-based model performed well-during the external validation. Moreover, the model satisfactorily performed in predicting short-term mortality in the external validation. These results indicate that the questionnaire-based ML model can estimate all-cause mortality with good discrimination for Chinese. Compared with previous death prediction models, our prediction model comes from the general Chinese population and improves the prediction ability of questionnaire variables through machine learning algorithm, which may be more suitable for application in primary health care of China.

### Strengths and limitations

Our study has several strengths. First, although a few ML prediction studies have been conducted on all-cause mortality, most of them considered death as a binary outcome rather than a time-to-event outcome. Our study considered the censoring of death, which reduced the miscalibration of the models. Second, all three approaches mainly used for predicting survival events, namely, penalized regression, boosting, and tree or forest, were included in this study. Furthermore, we comprehensively compared ML methods with traditional ones. Third, owing to the characteristics of the CHNS data, many variables for Chinese, such as lifestyle, activities, and health status, are considered in the process of developing the model. Therefore, the prediction model may be more suitable for Chinese. Fourth, to be more convenient to use, we developed a simple prediction model with only 20 questionnaire variables, and the performance of this model was not significantly different from those of the models with all variables. Fifth, although the development of our model was based on 6-year mortality data, three external validation cohorts were used to assess the predictive effectiveness of the model on long- and short-term mortalities, which showed that the model has good application potential for Chinese. Finally, we created a webpage for the ML-based model to enhance its application.

The present study has some shortcomings. First, although we included as many candidate factors as possible in the CHNS, several factors that cause important death burdens, such as environmental factors, were not included because of the research design of the CHNS. Second, there were no causes of death in this study due to data limitations. The issue on whether there are differences in the prediction of different types of death by the prediction model still needs to be further explored. Third, because we excluded pregnant and nursing women and individuals with myocardial infarction, stroke, or tumor during the model development, our model is only applicable to the general population of China. Although our model has been validated in the CHARLS cohort of people over 45 years of age, further study is still necessary to determine whether the model is applicable to other special populations. Fourth, this model has not been verified based on data in other countries; therefore, it is not certain whether it can be applied to other populations. Fifth, the cause of death in our study including accidents or unnatural death which may add a degree of randomness.

## Conclusions

In this prospective population-based study, ML algorithms were shown to improve traditional approaches used for creating all-cause mortality prediction tools, and the model based on RSF analysis performed best among all models. Furthermore, no significant difference between the prediction performance of the questionnaire-based ML model, which only included 20 variables, and that of the model with all variables that included laboratory variables was found. The simple questionnaire-based ML prediction model, which needs to be further explored, is of great importance for its accuracy and suitability to the Chinese general population.

## Data availability statement

The datasets presented in this study can be found in online repositories. The data and research materials supporting the findings of this study can be found on the CHNS official website (http://www.cpc.unc.edu/projects/china) and CHARLS official website (http://charls.pku.edu.cn/).

## Ethics statement

The studies involving human participants were reviewed and approved by the Institutional Review Boards of the University of North Carolina at Chapel Hill, National Institute of Nutrition and Food Safety, and Chinese Center for Disease Control and Prevention. The patients/participants provided their written informed consent to participate in this study.

## Author contributions

HZ and YL had full access to all of the data in the study and takes responsibility for the integrity of the data and the accuracy of the data analysis. HZ, ZL, and YL planned the concept of this study. HZ, ZL, NY, and YL conducted data extraction and analysis. LH, JW, FP, and WL carried out data cleaning and material support. All authors critically reviewed, revised, and contributed to the final manuscript.
