# Supplementary material for: Development and validation of questionnaire-based machine learning models for predicting all-cause mortality in a representative population of China
Source: Front Public Health. 2023 Jan 27;11:1033070. doi: 10.3389/fpubh.2023.1033070 (PMC9911458; doi:10.3389/fpubh.2023.1033070)

Development and Validation of Questionnaire-based Machine Learning Models for Predicting All-Cause Mortality in a Representative Population of China

Ziyi Li, Na Yang, Liyun He, Jialu Wang, Fan Ping, Wei Li, Lingling Xu, Huabing Zhang*, Yuxiu Li*

Supplementary Material

Contents

Description of cohorts

Description of machine learning algorithms

eTable 1. List of predictor variables in the CHNS cohort, including descriptive statistics.

eTable 2 Duration years of diseases at Baseline.

eTable 3. Performance of the Models for Predicting All-cause Mortality in training cohort.

eTable 4. Sensitivity analyses: Performance of the Models for Predicting 9-Year Risk of Mortality in the CHNS 2006-2015 Survey

eTable 5. Sensitivity analyses: Performance of the Models for Predicting 11-Year Risk of Mortality in the CHNS 2004-2015 Survey

eTable 6. The list of 20 variables for the online questionnaire-based prediction model.

eFigure 1. Study population flowchart

eFigure 2. Calibration of the models with all variables for predicting 6-year all-cause mortality in internal validation cohort

eFigure 3. Calibration of the models with variables excluding laboratory variables for predicting 6-year all-cause mortality in internal validation cohort

eFigure 4. Calibration of the models with laboratory variables for predicting 6-year all-cause mortality in internal validation cohort

eFigure 5. Top 20 Risk factors predicted by RSF analysis

eFigure 6. Calibration of the models with all variables for predicting 6-year all-cause mortality in internal validation cohort

eFigure 7. Examples of predicted 6-year risk of mortality using the online ML models.

eFigure 8. Examples of predicted 6-year risk of mortality using the online ML model with 10 variables.

eFigure 9. Calibration of the questionnaire-based ML model for predicting mortality in CHARLES.

eFigure 10. Calibration of the questionnaire-based ML model for predicting mortality in CHNS 2006-2015 data

eFigure 11. Calibration of the questionnaire-based ML model for predicting mortality in CHNS 2004-2015 data

Description of cohorts

The China Health and Nutrition Survey (CHNS):

The CHNS was conducted to study the influence of social and economic transformation on the health and nutritional status of a representative population of China. Briefly, the CHNS is a national, open cohort study that started in 1989 and has been followed up every two to four years. It is a longitudinal study which covered 15 provinces with significant differences in geography, economic development, and public resources. A multistage random clustering method was used to draw samples. First, a weighted sampling scheme was employed to randomly select four counties in each province after stratifying them by income. Then, villages and townships within the counties and urban and suburban neighborhoods within the cities were randomly selected. The study was approved by the Institutional Review Boards of the University of North Carolina at Chapel Hill, National Institute of Nutrition and Food Safety, and Chinese Center for Disease Control and Prevention. All participants provided their written informed consent.

The investigation contained information on individuals, households, and communities. Trained staff administered a standardized questionnaire to collect information on individuals, households, and communities. Trained clinicians performed physical examinations, including measurements of height, weight, and blood pressure. Blood samples were collected from an empty stomach after the participants maintained a regular pattern of life for at least three days.

The China Health and Retirement Longitudinal Study (CHARLS)

The CHARLS is a nationally representative longitudinal survey of the social, economic, and health circumstances of persons in China 45 years of age or older. The national baseline survey for the study was started in 2011 and has been followed up every two years. Physical measurements are made at every follow-up, and blood sample collection is done once in every two follow-up periods. This initial sample covered 10,257 households in 150 county-level units and 450 villages or urban communities across 28 provinces. Samples were chosen through multistage probability sampling.

Description of machine learning algorithms:

In the development of the ML models to predict time-to-event survival outcomes, three main approaches were used: penalized regression, boosting, and tree or forest. LASSO was selected for the penalized regression algorithm. As a generalized linear model that fits with the penalized maximum likelihood, LASSO provides a variable selection approach by penalizing coefficients and produces the best-fitting parsimonious model by cross-validation.

For the boosting algorithm, the glmBoost and GBM methods were utilized. The glmBoost method provides feature selection and reduces the model complexity for time-to-event analysis. However, it can only detect linear relationships between variables. GBM is a powerful ensemble machine learning method that uses a forward stage-wise strategy to build an additive model .

For the tree and forest algorithms, survival tree, RSF, and CIF were employed. Survival tree analysis provides parsimonious, transparent decision rules that maximize predictive accuracy and evaluates model cross-generalizability. RSF is an ensemble forest method for the analysis of right-censored data. An ensemble survival probability is estimated by averaging over all trees in a forest.

The CIF model can reduce biases in covariate selection by separating the algorithm for selecting the best covariate.

Supplementary Table 1 List of predictor variables in the CHNS cohort, including descriptive statistics.

Variable	Statistic
**Demographics**	
Age	50.18 (14.65)
Men	4004(47.92%)
Registered residence type: urban	2713(32.47%)
Minority nationality	1016(12.16%)
Region of residence: Northern	3631(43.46%)
**Family relations**	
Marital status	
Single	628 (7.52%)
Married	6954 (83.23%)
Divorced	773(9.25%)
Father lives at home	785(9.40%)
Mother live at home	977(11.70%)
Number of family members	4 (2)
**Community score**	
Quality of Health Score	5.95(2.57)
Housing Component Score	7.57(2.01)
Market Component Score	4.83(3.49)
Social Services Score	3.67(3.13)
Transportation Component Score	5.89(2.18)
Community Education Category	3.44(1.40)
Modern Markets Component Score	4.36(2.91)
Sanitation Score	6.80(2.92)
Urbanization Index	67.20(19.43)
Communications Component Score	6.78(1.55)
Diversity Score	5.41(1.09)
Economic Component Score	6.57(3.24)
**Activity**	
Housework: Buying food	5217(62.44%)
Time spending on buying food per day (minutes)	20(30)
Housework: Preparing food	5088(60.90%)
Time spending on Preparing food per day (minutes)	30(60)
Housework: Washing clothes	4688(56.11%)
Time spending on washing per day (minutes)	20(30)
Housework: Cleaning house	5118(61.26)
Time spending on cleaning per day (minutes)	10(30)
Total housework time (minutes)	90(80)
Time of working in a week (hours)	20(20)

Reading	2065(24.70%)
Playing video game	117(1.43%)
Playing computer game	674(8.1%)
Surfing the internet	922(11.00%)
Activity at watching movie	657(7.9%)
TV time in a week (hours)	14(14)
Sitting time in a week (hours)	14(12.91)
Field activity	265(3.20%)
Gymnastics	166(2.00%)
Soccer	114(1.41%)
Badminton	150(1.82)
Other activity	244(2.94%)
Activity time, (minutes)	237(56)
Activity level	
Low	4237 (50.71%)
Moderate	1241 (14.85%)
High	2871 (34.43%)
Bedtime (hours)	8(1)
**Socioeconomic**	
Highest Level of Education Attained	
None	1919(22.97%)
Grad from primary	1648(19.72%)
Lower middle school degree	2791(33.41%)
Upper middle school degree	992(11.87%)
Technical or vocational degree	595(7.12%)
University or college degree	406(4.86%)
Master's degree or higher	4(0.05%)
Education: senior high school or above	
Higher than senior high school	1005(12.02)
Above senior high school	7350(87.98)
Per capita Household income inflated to 2015 (Thousand yuan)	32.22(38.80)
Total net household income inflated to 2015 (Thousand yuan)	35.38(40.58)
Total gross household income inflated to 2015 (Thousand yuan)	9.45(11.7)
Individual income per year (Thousand yuan)	13.81(14.15)
Working	5003(59.88%)
Having a second occupation	53(6.38%)
Having insurance	7610 (91.09%)
Type of insurance	
Urban resident basic	800 (9.57%)
Urban employee basic	1356 (16.23%)
Cooperative insurance	4846 (58.00%)
Commercial	207 (2.48%)

Free medical	327 (3.91%)
Other	74 (0.89%)
Do not have an insurance	745 (8.91%)
**Macronutrient intakes and dietary behaviors**	
Carbohydrate (g)	283.32(129.72)
Fat (g)	69.00(44.72)
Protein (g)	62.87(28.31)
Energy (kcal)	2081.38(827.65)
Frequency of breakfast (%)	0.88(0.36)
Frequency of lunch (%)	0.85(0.33)
Frequency of dinner, (%)	0.94(0.21)
Frequency of snack (%)	0.27(0.38)
Proportion of breakfasts (%)	0.23(0.08)
Proportion of supper (%)	0.39(0.06)
Proportion of supper (%)	0.38(0.08)
Proportion of breakfast at home (%)	0.82(0.36)
Proportion of lunch at home (%)	0.85(0.32)
Proportion of supper at home (%)	0.94(0.21)
Food likes: fast food	
Strongly disagree	896 (10.72%)
Somewhat disagree	5936 (71.04%)
Neutral	959 (11.48%)
Somewhat agree	520 (6.22%)
Strongly agree	44 (0.57%)
Food likes: salty snack food	
Strongly disagree	676 (8.10%)
Somewhat disagree	5668 (6.18%)
Neutral	1235 (14.78%)
Somewhat agree	748 (8.95%)
Strongly agree	28 (0.33%)
Food likes: fruits	
Strongly disagree	45(0.54%)
Somewhat disagree	386(4.62%)
Neutral	1395(16.70%)
Somewhat agree	6150(73.60%)
Strongly agree	379(4.54%)
Food likes: vegetables	
Strongly disagree	30(0.36%)
Somewhat disagree	106(1.27%)
Neutral	861(10.31%)
Somewhat agree	6876(82.30%)
Strongly agree	482(5.77%)
Food likes: soft/sugared drinks	
Strongly disagree	396(4.74%)
Somewhat disagree	4146(49.62%)
Neutral	2238(26.78%)

Somewhat agree	1519(18.14%)
**Lifestyle**	
Drinking soft water	2832 (33.90%)
Bottles of water intake per day	4 (2)
Smoking	2691 (32.21%)
Number of cigarettes smoked per day	5 (7)
Drinking Tea	2975 (35.61%)
Frequency of drinking tea	
Every day	2112 (25.28%)
4-5 times a week	227 (2.72%)
2-3 times a week	411 (4.92%)
No more than once a week	109 (1.30%)
2-3 times in past 30 days	57 (0.68%)
Only once in past 30 days	18 (0.22%)
None in past 30 days	5421(64.88%)
Drinking Coffee	201 (2.41%)
Frequency of drinking coffee	
Every day	23(0.27%)
2-3 times a week	21(0.25%)
No more than once a week	38(0.45%)
2-3 times in past 30 days	45(0.54%)
Only once in past 30 days	22(0.25%)
None in past 30 days	8186(97.98%)
Drinking	2808 (33.61%)
Frequency of drinking	
Every day	791(9.5%)
2-3 times a week	400(4.80%)
No more than once a week	675(8.10%)
2-3 times in past 30 days	583(7.00%)
Only once in past 30 days	359(4.30%)
None in past 30 days	5547(66.40%)
**Knowledge**	
Know about Chinese dietary guidelines	1082 (12.95%)
Diet knowledge: lots of fruit/vegetables	
Strongly disagree	51(0.61%)
Somewhat disagree	943(11.29%)
Neutral	845(10.11%)
Somewhat agree	6102(73.03%)
Strongly agree	414(4.96%)
Diet knowledge: lots of sugar	
Strongly disagree	293(3.51%)
Somewhat disagree	6521(78.05%)
Neutral	945(11.31%)
Somewhat agree	562(6.73%)
Strongly agree	34(0.41%)
Diet knowledge: variety of foods	

Strongly disagree	23(0.27%)
Somewhat disagree	522(6.25%)
Neutral	1115(13.35%)
Somewhat agree	6451(77.21%)
Strongly agree	244(2.92%)
Diet knowledge: diet high in fat	
Strongly disagree	428(5.12%)
Somewhat disagree	6359(76.11%)
Neutral	859(10.28%)
Somewhat agree	656(7.85%)
Strongly agree	53(0.63%)
Diet knowledge: lots of staple food	
Strongly disagree	67(0.80%)
Somewhat disagree	2841(34.00%)
Neutral	1966(23.53%)
Somewhat agree	3428(41.03%)
Strongly agree	58(0.69%)
Diet knowledge: lots of animal products	
Strongly disagree	171(2.05%)
Somewhat disagree	4539(54.33%)
Neutral	1243(14.88%)
Somewhat agree	2319(27.76%)
Strongly agree	83(0.99%)
Diet knowledge： amt animal meat/fat	
Strongly disagree	50(0.60%)
Somewhat disagree	897(10.74%)
Neutral	958(11.47%)
Somewhat agree	6223(74.48%)
Strongly agree	227(2.72%)
Diet knowledge: milk &dairy products	
Strongly disagree	30(0.36%)
Somewhat disagree	164(1.96%)
Neutral	658(7.88%)
Somewhat agree	7084(84.79%)
Strongly agree	419(5.01%)
Diet knowledge: beans&bean products	
Strongly disagree	35(0.42%)
Somewhat disagree	103(1.23%)
Neutral	568(6.80%)
Somewhat agree	7182(85.96%)
Strongly agree	467(5.59)
Diet knowledge: physical activities	
Strongly disagree	37(0.44%)
Somewhat disagree	277(3.32%)
Neutral	918(10.99%)
Somewhat agree	6712(80.33%)
Strongly agree	411(4.92%)

Diet knowledge: intense physical act	
Strongly disagree	83(0.99%)
Somewhat disagree	2165(25.91%)
Neutral	1393(16.67%)
Somewhat agree	4585(54.87%)
Strongly agree	129(1.54%)
Diet knowledge: heavier body	
Strongly disagree	1034(12.37%)
Somewhat disagree	6352(76.03%)
Neutral	561(6.71%)
Somewhat agree	332(3.97%)
Strongly agree	76(0.91%)
Activity likes: walking, Tai chi	
Dislike very much	281(3.36%)
Dislike somewhat	3834(45.89%)
Neutral	1723(20.62%)
Like somewhat	2342(28.03%)
Like very much	175(2.09%)
Activity likes: sports	
Dislike very much	319(3.82%)
Dislike somewhat	5543(66.34%)
Neutral	1669(19.98%)
Like somewhat	764(9.14%)
Like very much	60(0.71%)
Activity likes: body building	
Dislike very much	276(3.30%)
Dislike somewhat	5021(60.10%)
Neutral	1934(23.15%)
Like somewhat	1072(12.83%)
Like very much	52(0.62%)
Activity likes: watching TV	
Dislike very much	104(1.24%)
Dislike somewhat	597(7.15%)
Neutral	1559(18.66%)
Like somewhat	5757(68.90%)
Like very much	338(4.05%)
Activity likes: computer/video games	
Dislike very much	612(7.32%)
Dislike somewhat	5671(67.88%)
Neutral	1025(12.27%)
Like somewhat	920(11.01%)
Like very much	127(1.52%)
Activity likes: reading	
Dislike very much	349(4.18%)
Dislike somewhat	4453(53.30%)
Neutral	1916(22.93%)

Like somewhat	1535(18.37%)
Like very much	102(1.22%)
Priorities: good income	
Not important at all	87(1.04%)
Not very important	311(3.72%)
Important	4532(54.25%)
Very important	2622(31.38%)
Most important	803(9.61%)
Priorities: physically active	
Not important at all	136(1.62%)
Not very important	917(10.98%)
Important	5133(61.44%)
Very important	1946(23.29%)
Most important	223(2.67%)
Priorities: healthy diet	
Not important at all	57(0.68%)
Not very important	427(5.11%)
Important	5335(63.85%)
Very important	2249(26.91%)
Most important	287(3.44%)
Priorities: child insist on physical exercise	
Not important at all	45(0.54%)
Not very important	230(2.75%)
Important	5025(60.14%)
Very important	2583(30.92%)
Most important	472(5.65%)
Priorities: Children adhere to a healthy diet	
Not important at all	37(0.44%)
Not very important	160(1.92%)
Important	4938(59.10%)
Very important	2620(31.36%)
Most important	600 (7.18%)
**Health status**	
Hypertension	997(11.93%)
Diabetes	231(2.76%)
Fracture	376(4.50%)
Heart disease	62 (0.74%)
Respiratory disease	277(3.31%)
Digest disease	106(1.27%)
Muscle disease	61(0.7%)
Joint disease	408 (4.88%)
Infectious diseases	100 (1.20%)
Noncommunicable diseases	361(4.32%)
Other disease	64(0.77%)
Illness last month	1243(14.20%)
Fever	620 (7.42%)

Diarrhea	116 (1.39%)
Asthma	64 (0.77%)
Stomachache	213 (2.55%)
Headache	405 (4.85%)
Chest pain	117(1.40%)
Visit a folk doctor	366(4.33%)
Seek formal medical care	89 (1.07%)
**Physical examination**	
DBP	82 (13)
SBP	124 (19)
BMI	23.31 (3.43)
Waist circumference (cm)	83.18 (10.29)
Hip circumference (cm)	94.28 (8.03)
Upper arm circumference (cm)	27.29 (4.79)
Triceps skin fold (cm)	12.26 (7.68)
**Laboratory examinations**	
Red Blood Cell Count	4.72 (0.64)
White Blood Cell Count	6.30 (1.81)
Platelet Count	212.73 (63.21)
Hemoglobin (g/L)	14.20 (1.95)
Total Blood Protein (g/L)	74.95 (4.94)
Albumin (g/L)	45.24 (3.18)
Total Cholesterol (mmol/L)	4.87 (0.96)
Triglycerides (mmol/L)	1.56 (1.28)
High-Density Lipoprotein Cholesterol (mg/dL)	1.43 (0.47)
Low-Density Lipoprotein Cholesterol (mg/dL)	3.01 (0.92)
Apolipoprotein A (g/L)	1.16 (0.39)
Apolipoprotein B (g/L)	0.91 (0.25)
Alanine Aminotransferase (U/L)	24.17 (19.41)
Creatinine	87.28 (20.36)
Urea (mmol/L)	5.44 (1.45)
Uric Acid (mg/dL)	306.78 (99.51)
Glucose (mmol/L)	5.34 (1.35)
Hemoglobin A1c (%)	5.60 (0.86)
Insulin (μIU/ml)	13.88 (21.46)
High-Sensitivity CRP (mg/dL)	2.32 (7.93)
Magnesium (mmol/L)	0.93 (0.10)
Ferritin (ng/ml)	136.48 (176.14)
Transferrin (mg/L)	1.46 (0.68)

Mean ± standard deviation (SD) or median with inter-quartile range (IQR) were calculated for continuous variables. Totals and percentages were calculated for categorical variables.

eTable 2 Duration years of diseases at Baseline.

	Died	Alive
**Duration years of Diabetes**	7 ± 3	8 ± 3
**Duration years of hypertension**	8 ± 4	4 ± 2

Median with inter-quartile range (IQR) were calculated

eTable 3 Performance of the Models for Predicting All-cause Mortality in training cohort.

		COX	Lasso	glmBoost	ST	RSF	CIF	GBM
**All**	**C-index**	0.80	0.87	0.83	0.87	0.86	0.92	0.87
		(0.72-0.88)	(0.80-0.94)	(0.76-0.92)	(0.78-0.96)	(0.80-0.92)	(0.85-0.99)	(0.79-0.95)
	**Time-ROC**	0.84	0.89	0.80	0.83	0.85	0.93	0.86
	**Brier**	0.10	0.08	0.08	0.08	0.08	0.07	0.07
**Without laboratory variables**	**C-index**	0.80	0.84	0.85	0.85	0.86	0.91	0.82
		(0.72-0.88)	(0.77-0.91)	(0.75-0.95)	(0.75-0.95)	(0.80-0.92)	(0.82-0.99)	(0.74-0.90)
	**Time-ROC**	0.83	0.82	0.83	0.86	0.83	0.89	0.79
	**Brier**	0.11	0.11	0.10	0.08	0.08	0.08	0.09
**Laboratory variables**	**C-index**	0.68	0.66	0.69	0.66	0.74	0.72	0.69
		(0.56-0.80)	(0.56-0.76)	(0.59-0.79)	(0.57-0.75)	(0.65-0.83)	(0.63-0.81)	(0.59-0.79)
	**Time-ROC**	0.65	0.66	0.68	0.63	0.74	0.68	0.63

**Brier**	0.14	0.15	0.15	0.15	0.13	0.14	0.14

Abbreviations: COX, Cox proportional hazards regression model; Lasso, least absolute shrinkage and selection operator regression model; glmBoost, boosted generalized linear model; ST, survival tree model; CIF, conditional inference forest model; RSF, random forest survival analysis model; GBM, gradient boosting model. All: Models with all variables; Without laboratory variables: Models with variables excluding laboratory variables; Laboratory variables: Models with only laboratory variables.

eTable 4 Sensitivity analyses: Performance of the Models for Predicting 9-Year Risk of Mortality in the CHNS 2006-2015 Survey.

		COX	Lasso	ST	RSF	CIF	glmBoost	GBM
**Training**	**C-index**	0.80	0.83	0.88	0.89	0.90	0.86	0.85
		(0.75-0.85)	(0.81-0.85)	(0.85-0.91)	(0.86-0.94)	(0.87-0.93)	(0.81-0.91)	(0.81-0.89)
	**Time-ROC**	0.82	0.85	0.08	0.90	0.89	0.83	0.86
	**Brier**	0.07	0.06	0.10	0.04	0.08	0.08	0.07
**Internal Validation**	**C-index**	0.79	0.84	0.84	0.85	0.85	0.82	0.84
		(0.74-0.84)	(0.82-0.88)	(0.81-0.87)	(0.82-0.88)	(0.82-0.88)	(0.78-0.86)	(0.80-0.88)
	**Time-ROC**	0.79	0.86	0.85	0.86	0.86	0.81	0.84
	**Brier**	0.07	0.07	0.06	0.04	0.08	0.08	0.07

Abbreviations: COX, Cox proportional hazards regression model; Lasso, least absolute shrinkage and selection operator regression model; glmBoost, boosted generalized linear model; ST, survival tree model; CIF, conditional inference forest model; RSF, random forest survival analysis model; GBM, gradient boosting model.

eTable 5 Sensitivity analyses: Performance of the Models for Predicting 11-Year Risk of Mortality in the CHNS 2004-2015 Survey.

		COX	Lasso	ST	RSF	CIF	glmBoost	GBM
**Training**	**C-index**	0.80	0.84	0.80	0.85	0.87	0.86	0.87
		(0.76-0.84)	(0.80-0.88)	(0.75-0.85)	(0.80-0.90)	(0.82-0.92)	(0.81-0.91)	(0.81-0.93)
	**Time-ROC**	0.80	0.83	0.80	0.85	0.85	0.83	0.89
	**Brier**	0.13	0.08	0.10	0.04	0.04	0.08	0.05
**Internal Validation**	**C-index**	0.77	0.81	0.80	0.83	0.82	0.82	0.82
		(0.72-0.82)	(0.78-0.84)	(0.77-0.83)	(0.80-0.86)	(0.79-0.85)	(0.78-0.86)	(0.79-0.85)
	**Time-ROC**	0.78	0.82	0.79	0.83	0.79	0.81	0.80
	**Brier**	0.091	0.048	0.10	0.06	0.08	0.08	0.08

Abbreviations: COX, Cox proportional hazards regression model; Lasso, least absolute shrinkage and selection operator regression model; glmBoost, boosted generalized linear model; ST, survival tree model; CIF, conditional inference forest model; RSF, random forest survival analysis model; GBM, gradient boosting model.

eTable 6. The list of 20 variables for the online questionnaire-based prediction model.

Variable	Measurement
Age	Integer number calculated by birth year and month.
Hypertension	Individual who has been diagnosed with hypertension by a doctor before or the blood pressure meets the diagnostic criteria.
SBP	The average of the three measurements of systolic blood pressure.
Asthma	Have you felt asthma in the last month.
Income	Individual income per year (Thousand yuan).
Smoking	Individuals still smoke cigarettes now.
Divorced	Marital status, select ‘No’ if not married.
BMI	Height divided by the square pf weight.
Upper arm circumference	Average of three measurements. (cm)
Time spends on buying food (minute)	Time spends on buying food per day.
Housework time	Time spends on doing housework per week. (minute)
Sleep time	Time spends on sleeping per day. (hour)
Hip circumference(cm)	Average of three measurements. (cm)
Sitting time	Time spends on sitting per day. (minute)
Work time	Time spends on working per week. (hour)
TV time	Time spends on watching TV per week. (minute)
Activity like： Watching TV	Options including dislike very much, dislike somewhat, neutral, like somewhat, like very much.
Breakfast proportion (%)	Proportion of breakfast in the whole day's energy.
Illness last month	Were you sick last month.
Highest Level of Education Attained	Options including none, grad from primary, lower middle school degree, upper middle school degree, technical or vocational degree, university or college degree, master's degree or higher.

eFigure 1 Study population flowchart.

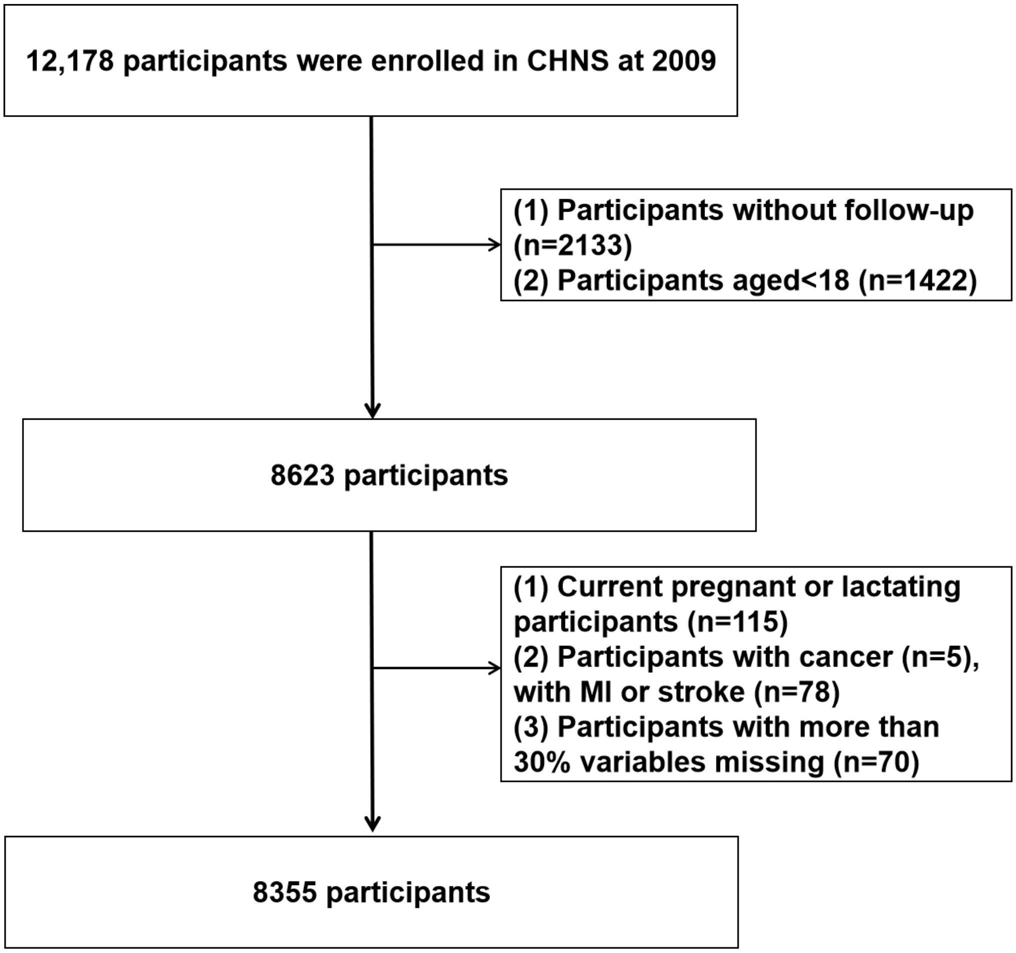

Abbreviations: MI, myocardial infarction.

eFigure 2 Calibration of the models with all variables for predicting 6-year all-cause mortality in internal validation cohort

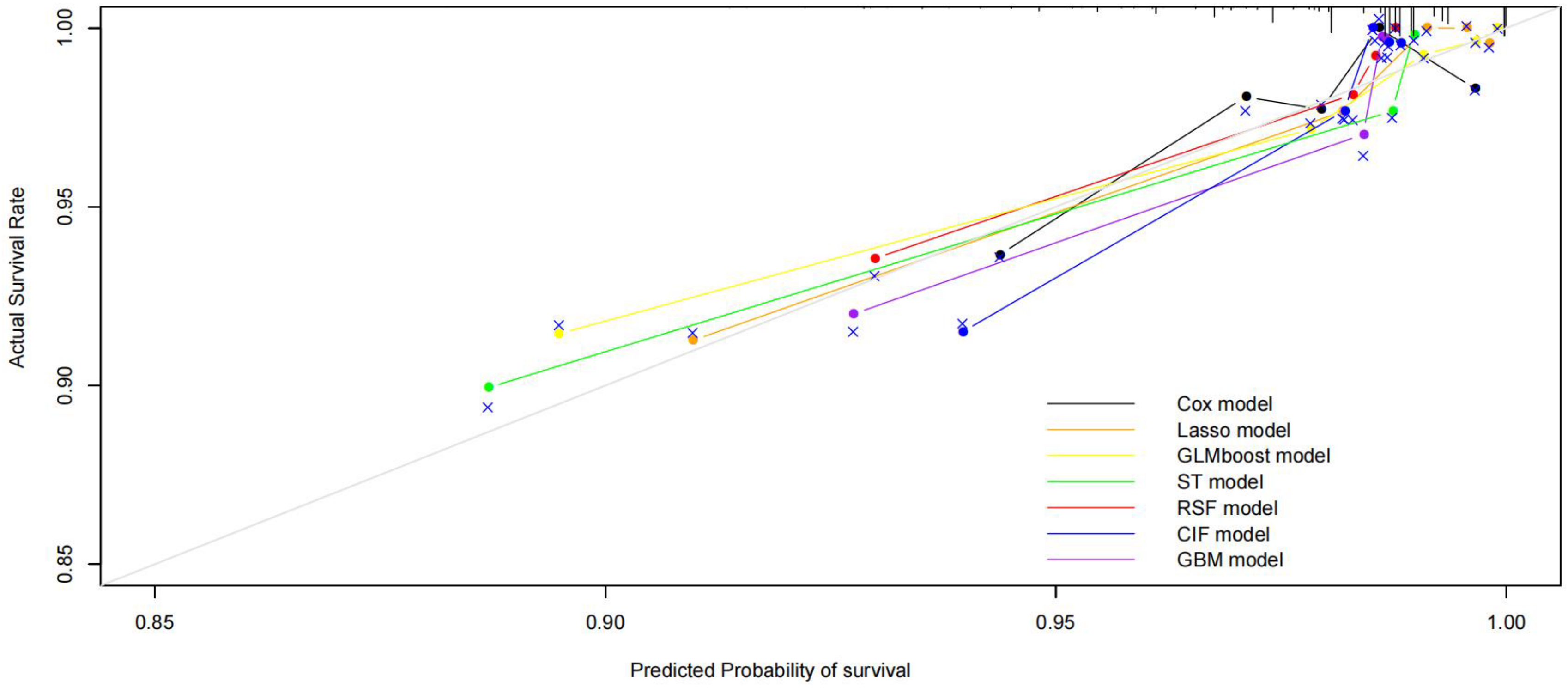

Abbreviations: COX, Cox proportional hazards regression model; Lasso, least absolute shrinkage and selection operator regression model; GLMBoost, boosted generalized linear model; ST, survival tree model; CIF, conditional inference forest model; RSF, random forest survival analysis model; GBM, gradient boosting model.

eFigure 3 Calibration of the models with variables excluding laboratory variables for predicting 6-year all-cause mortality in internal validation cohort.

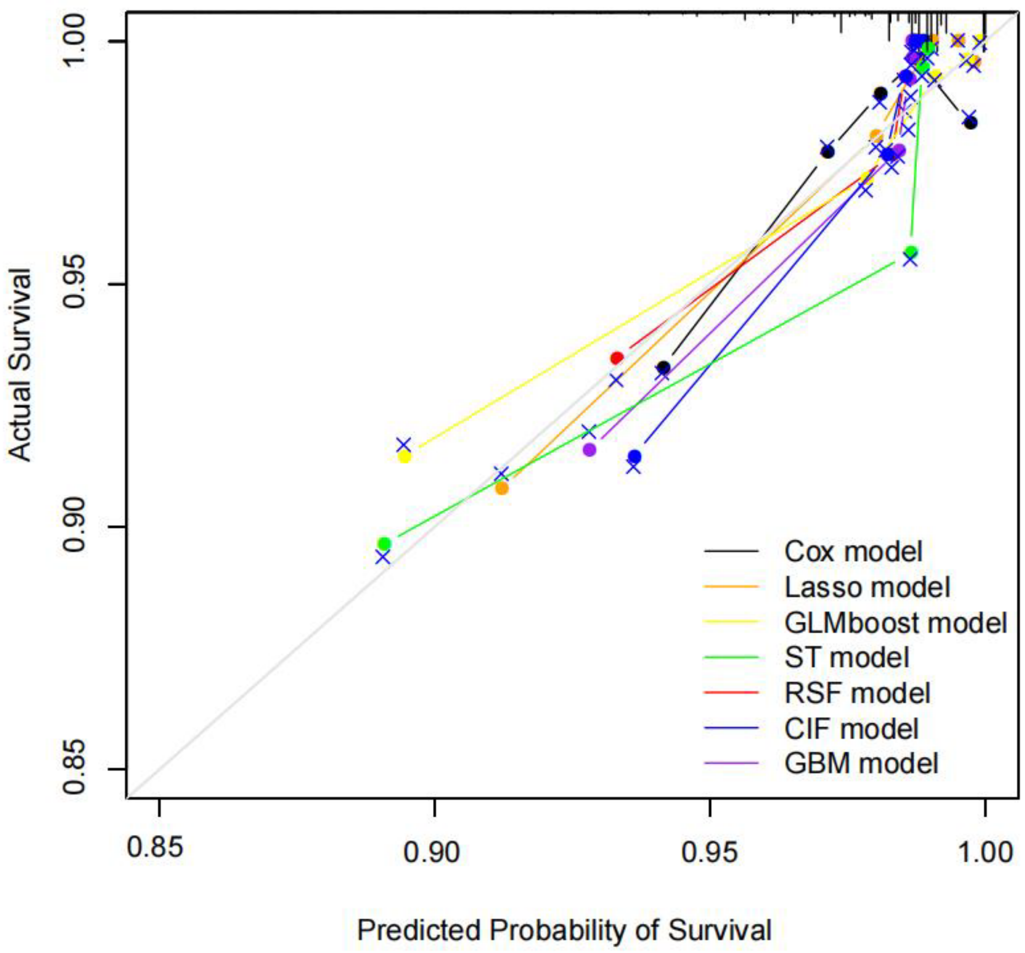

Abbreviations: COX, Cox proportional hazards regression model; Lasso, least absolute shrinkage and selection operator regression model; GLMBoost, boosted generalized linear model; ST, survival tree model; CIF, conditional inference forest model; RSF, random forest survival analysis model; GBM, gradient boosting model.

eFigure 4 Calibration of the models with laboratory variables for predicting 6-year all-cause mortality in internal validation cohort.

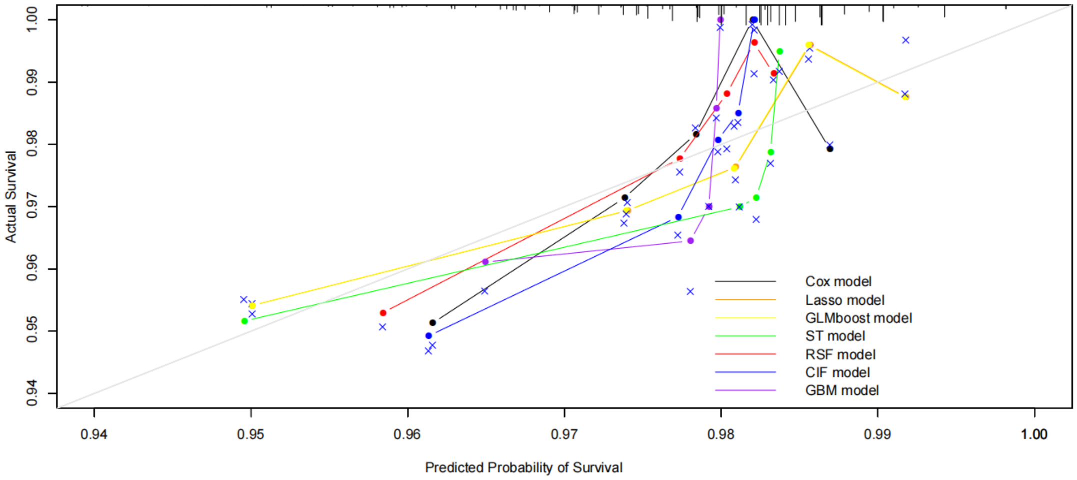

Abbreviations: COX, Cox proportional hazards regression model; Lasso, least absolute shrinkage and selection operator regression model; GLMBoost, boosted generalized linear model; ST, survival tree model; CIF, conditional inference forest model; RSF, random forest survival analysis model; GBM, gradient boosting model.

eFigure 5. Top 20 Risk factors predicted by RSF analysis

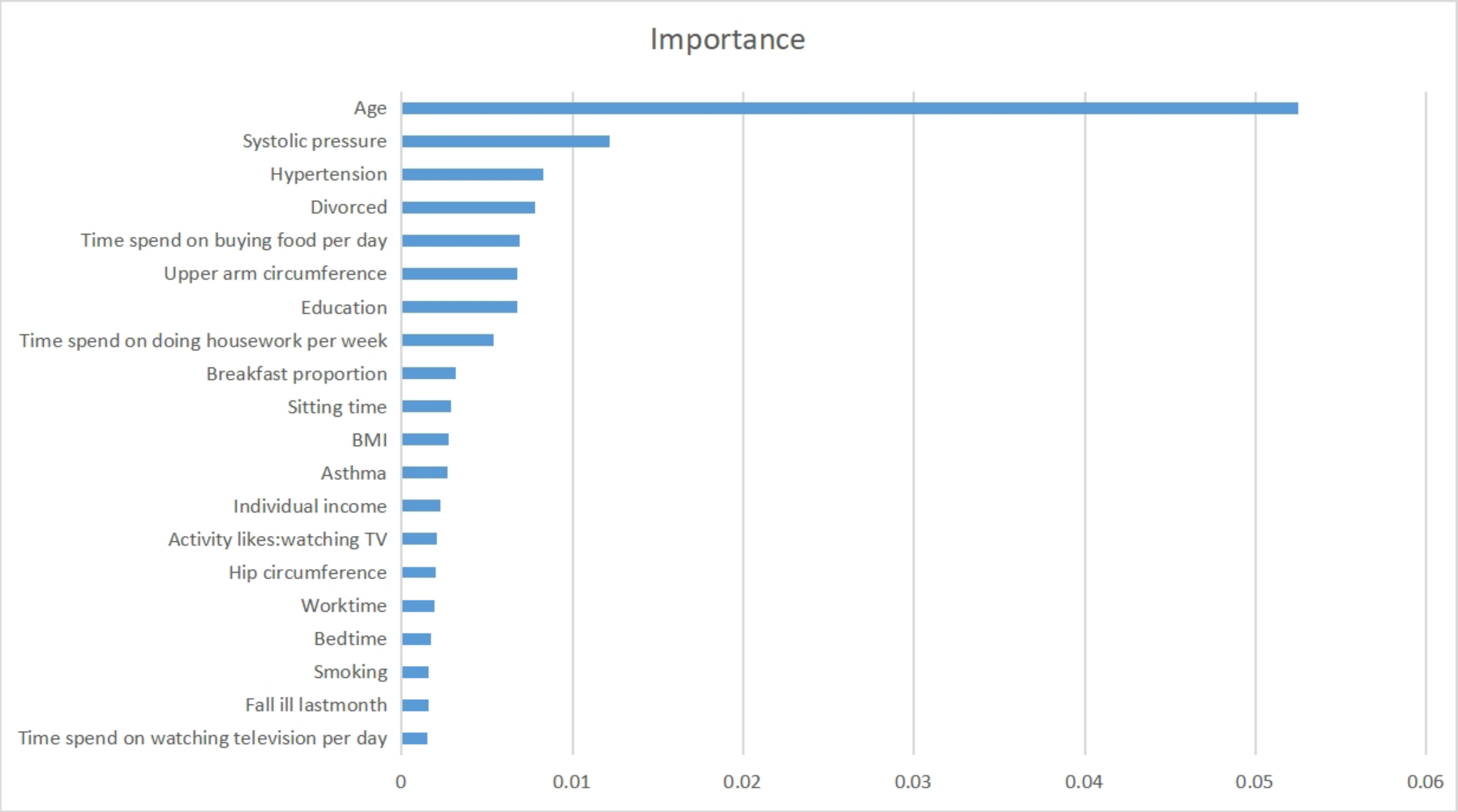

The importance of risk factors in the RSF model was identified. Abbreviations: RSF, random forest survival analysis model.

eFigure 6 Calibration of the RSF models for predicting 6-year all-cause mortality in internal validation cohort

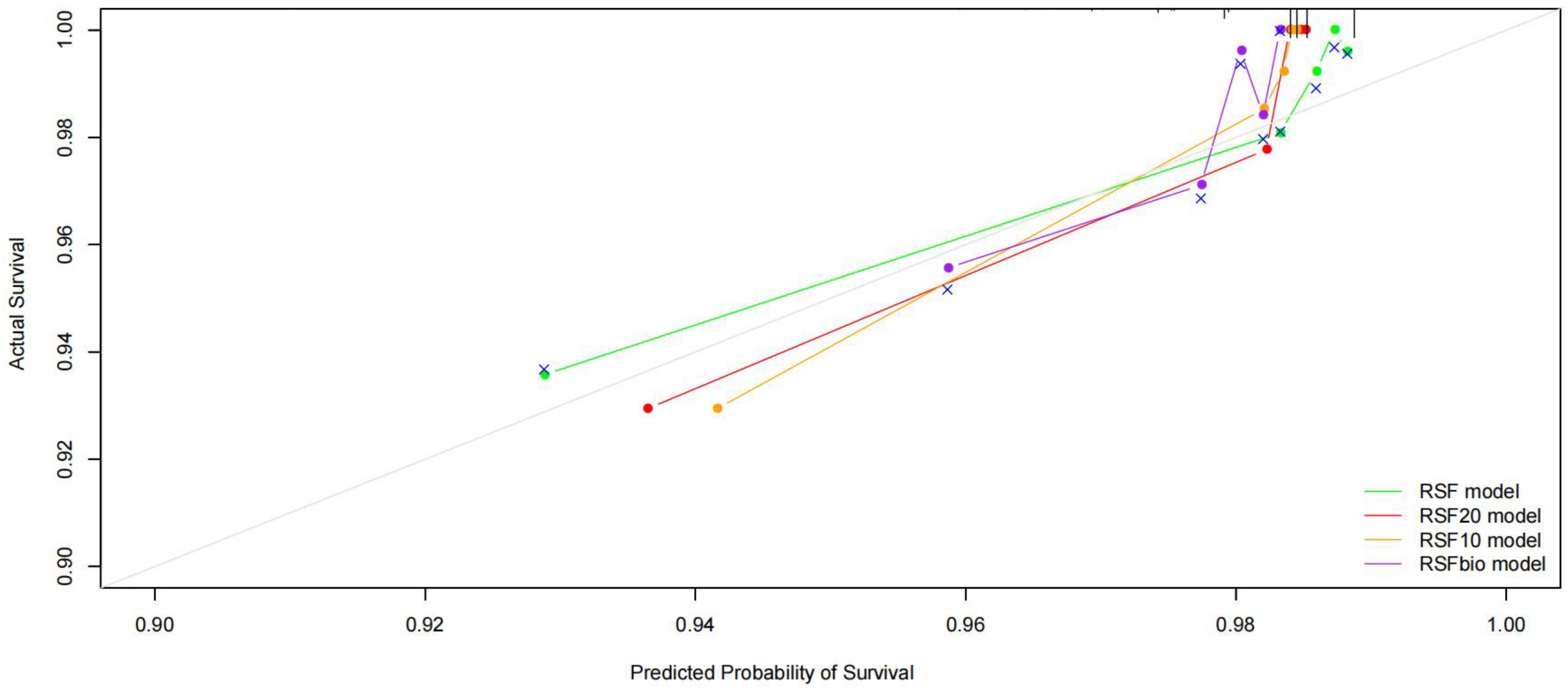

Abbreviations: RSF model：RSF with all 159 laboratory variables model, RSF20 model：RSF with self-reported predictors among top 20 variables model, RSF10 model：RSF with self-reported predictors among top 10 variables model, RSFbio model：RSF with only laboratory variables model.

eFigure 7. Examples of predicted 6-year risk of mortality using the online ML model with 20 variables.

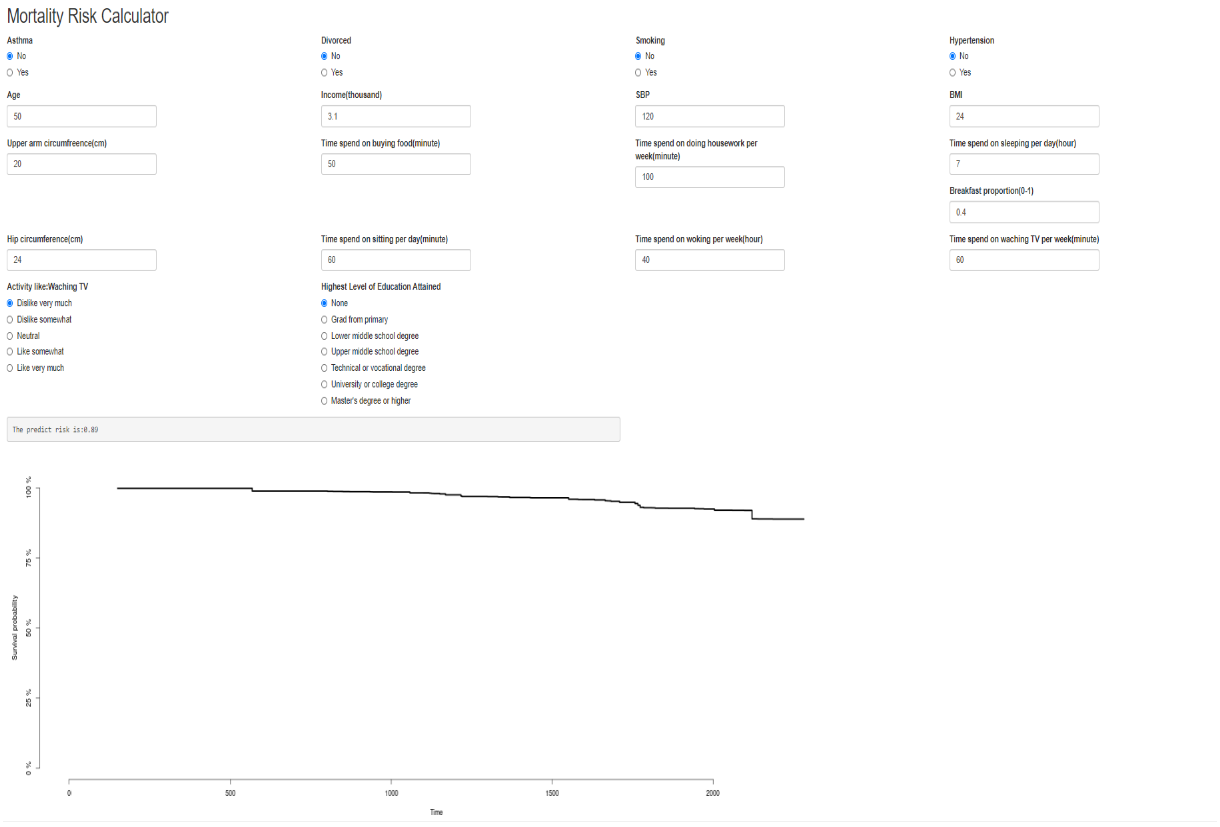

eFigure 8. Examples of predicted 6-year risk of mortality using the online ML model with 10 variables.（https://chnsmortalityprediction.shinyapps.io/mortality-10/）

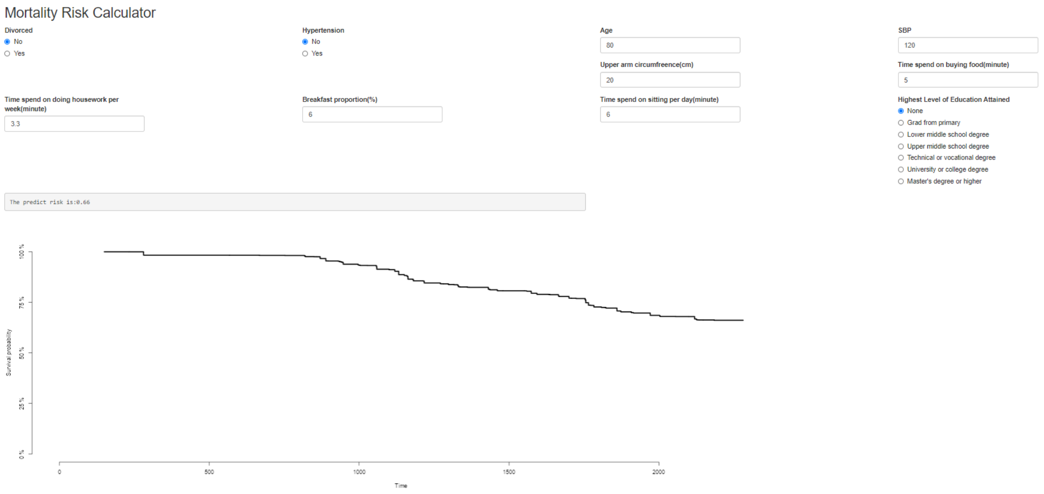

eFigure 9 Calibration of the questionnaire-based ML model for predicting mortality in CHARLES.

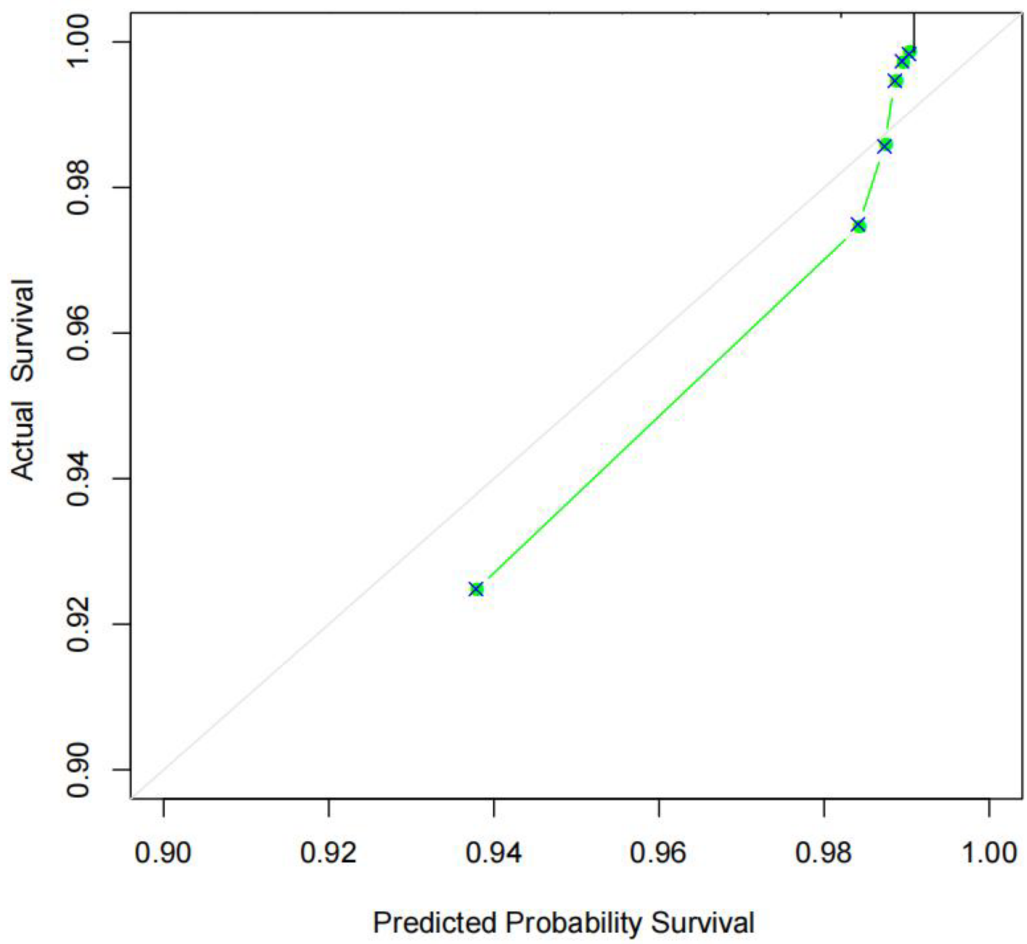

eFigure 10 Calibration of the questionnaire-based ML model for predicting mortality in CHNS 2006-2015 data.

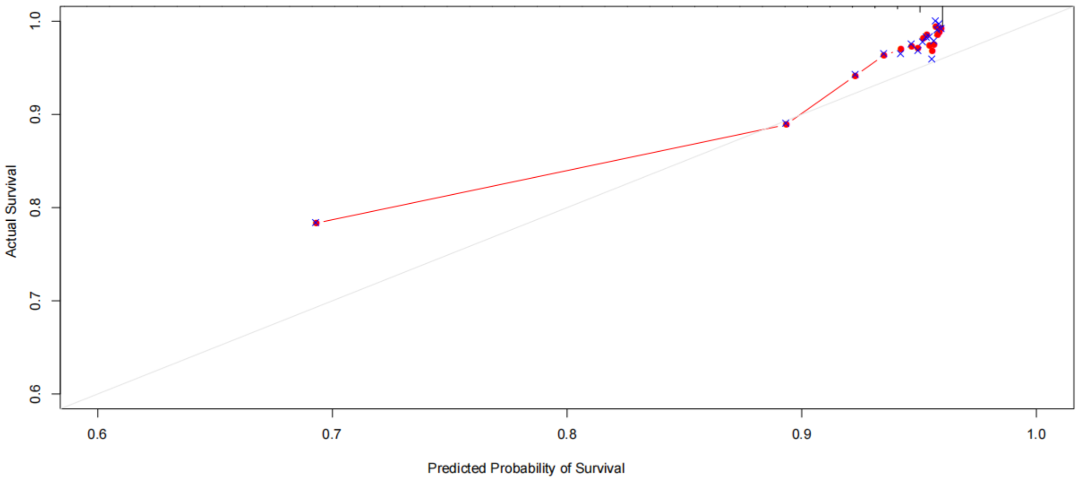

eFigure 11 Calibration of the questionnaire-based ML model for predicting mortality in CHNS 2004-2015 data.

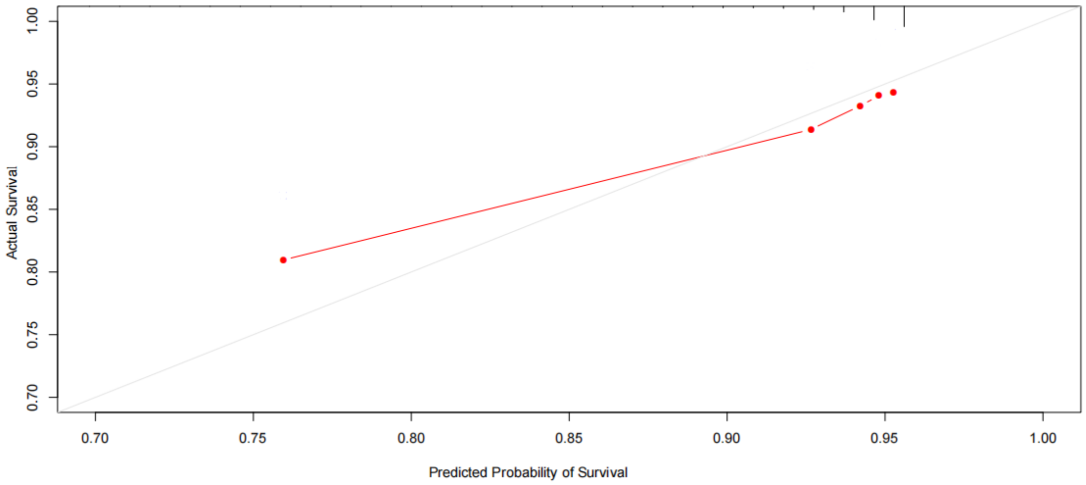

Supplement: Supplementary file 1 [file Data_Sheet_1.pdf]
